# Gastroesophageal reflux disease related to laparoscopic sleeve gastrectomy

**DOI:** 10.20517/2574-1225.2024.105

**Published:** 2025-08-26

**Authors:** Anisa Shaker, Edy Soffer

**Affiliations:** Swallowing and Esophageal Disorders Center, Division of Gastrointestinal and Liver Diseases, Department of Medicine, Keck School of Medicine of USC, University of Southern California, Los Angeles, CA 90089, USA.

**Keywords:** Gastroesophageal reflux disease, esophagitis, Barrett’s esophagus, obesity, laparoscopic, sleeve gastrectomy

## Abstract

Obesity remains a global public health burden. The most common surgical approach for this condition worldwide is laparoscopic sleeve gastrectomy (LSG). Although it is highly effective at achieving both short- and long-term weight loss, comparable to outcomes demonstrated by Roux-en-Y gastric bypass, there are growing concerns about the development or worsening of another prevalent and morbid condition, gastroesophageal reflux disease (GERD), following sleeve gastrectomy (SG). In this narrative review, we summarize current concerns related to GERD in the context of SG. We review the pathophysiologic mechanisms that predispose the SG anatomy to GERD, focus on the prevalence of *de novo* and worsening GERD and its associated complications, Barrett’s esophagus, review expert recommendations for GERD evaluation pre- and post-surgery, and discuss therapeutic options for those with severe GERD following SG.

## INTRODUCTION

Obesity is a global public health burden with growing prevalence and is a risk factor for a number of obesity-related health disorders, including malignancy, underscoring the urgent and ongoing need for effective interventions. Obesity is also a known risk factor for gastroesophageal reflux disease (GERD)^[1]^, a multifactorial disorder that is increasing in prevalence, affecting nearly 20% of the global population^[2]^, and present in nearly 51% of patients with severe obesity undergoing evaluation for bariatric surgery^[3]^.

While a number of surgical treatment options are available to treat obesity, the two most commonly performed procedures are sleeve gastrectomy (SG) and Roux-en-Y gastric bypass (RYGB), which account for 60% and 20% of anti-obesity surgeries, respectively. The adjustable gastric band, which has largely fallen out of favor due to intolerable side effects, and biliopancreatic diversion procedures, with or without duodenal switch, each account for less than 2% of cases^[4–6]^. Of note, several of the less common bariatric surgeries, such as biliopancreatic diversion with duodenal switch (BPD-DS) as well as newer approaches such as single-anastomosis duodeno-ileal bypass with SG (SADI-S), utilize SG as a first step or part of the procedure^[7]^.

Laparoscopic sleeve gastrectomy (LSG) is now one of the most commonly used bariatric procedures for weight loss. It involves a vertical gastric resection of approximately 75%−85% of the stomach along the greater curvature to the fundus, without any intestinal bypass. This procedure also removes the primary source of the hunger-inducing hormone, ghrelin, in the fundus. Candidates for LSG are individuals healthy enough to undergo surgery with a body mass index (BMI) ≥ 40, or a BMI ≥ 35 with at least one serious obesity-related health condition, e.g., type 2 diabetes mellitus (DM), obstructive sleep apnea, hypertension, arthritis, or hypercholesterolemia. Surgical intervention may also be considered for individuals with a BMI of 30–35 who have poorly controlled type 2 DM. Advantages of LSG include its relative simplicity, safety, and high efficacy in terms of weight loss and improvement of obesity-related health conditions, and quality of life. Compared to RYGB, it is a technically easier and faster operation, and in low-acuity patients, it may even allow for same-day discharge following the procedure^[8]^.

Although acute and chronic surgical complications of LSG, including bleeding, staple line leaks, and fistulae, have been reported, they are relatively infrequent^[9]^. Unlike RYGB, LSG carries no risk of internal hernias and does not preclude future endoscopic exploration of the duodenum or biliary tree, which may be necessary in cases such as choledocholithiasis^[10]^. Additional long-term risks, which can occur within months to several years after the procedure, include gastric stricture and vitamin or mineral deficiencies. Importantly, there are increased concerns that LSG may exacerbate preexisting GERD, contribute to the development of *de novo* GERD, or increase the risk of GERD-related complications such as Barrett’s esophagus (BE). GERD is a multifactorial disorder defined as the “presence of gastric contents in the esophagus that causes troublesome symptoms and/or injury to the esophageal mucosa”^[11]^. Postoperative GERD following LSG is an alarming concern, not only due to its associated morbidity^[12]^, but also because BE is a recognized risk factor for esophageal adenocarcinoma^[13]^. This review summarizes possible pathophysiologic mechanisms leading to GERD, the published prevalence of GERD after LSG, and strategies for preventing and treating GERD in the setting of LSG.

## PATHOPHYSIOLOGY OF GERD WITH LSG

A number of mechanisms have been proposed to play a role in the development of GERD symptoms after SG, in part through disruption of the “anti-reflux barrier”^[10]^. Changes in the angle of His during sleeve creation, a decrease in lower esophageal sphincter (LES) basal pressures due to resection of the low sling fibers^[14]^, and increased intragastric pressure within the narrow, tubular sleeve^[15]^ have all been proposed to create an imbalance between elevated gastric pressure and decreased LES pressure, potentially leading to postoperative reflux. Increased rates of hiatal hernia, with proximal sleeve migration above the hiatus due to dissection of the phreno-esophageal ligament, have also been proposed as a mechanism contributing to GERD development. Conversely, studies that report a decrease in GERD after SG suggest various pathophysiologic mechanisms including weight loss and associated decrease in intra-abdominal pressure, increased gastric emptying, and decreased acid production as a result of gastric fundus resection^[16]^. Both preclinical and clinical research indicate that the significantly altered anatomy following SG leads to accelerated gastric emptying^[17,18]^, thereby counteracting refluxogenic forces.

A decrease in gastric compliance following resection of the fundus may also contribute to increased gastric pressures after LSG. High-resolution impedance manometry (HRIM) post SG has shown that elevated intragastric pressure and impedance reflux events are frequent events^[19]^. A retrospective analysis of HRIM conducted a median of 11 months after SG in 53 patients found that increased intragastric pressure after water swallows occurred in 77% of cases, suggesting a reduction of gastric sleeve compliance. Despite this, esophageal bolus clearance remained intact in all cases. Impedance reflux episodes occurred in 53% of cases, of whom 40% reported typical GERD symptoms, compared to 5% among those without impedance reflux episodes. In addition, there was a higher frequency of ineffective esophageal motility. No correlation was found between increased intragastric pressure and impedance reflux episodes^[19]^, suggesting that elevated intragastric pressure is a manometric signature of SG rather than a causative factor in GERD.

Surgical technique may also play a role in the development of GERD after SG. Earlier studies showed a decrease in baseline LES pressures following SG without antral preservation, which was attributed to the division of LES sling fibers during transection of the gastric wall^[14]^. Interestingly, more recent studies show that hypotensive baseline LES pressures are not predictive of GERD after SG^[20]^. In a single-center retrospective study of 69 patients who underwent SG with antral preservation, high-resolution esophageal manometry and 24-hour pH monitoring were performed. Although LES length was reduced postoperatively (4.3 cm *vs*. 3.6 cm, *P* =0.00032), there were no significant changes in resting pre- and postoperative LES pressures. Moreover, the sensitivity, specificity, positive predictive value, and negative predictive value of a preoperative baseline hypotensive LES in predicting GERD (defined as a Demeester score > 14.72, regardless of the presence of GERD symptoms) were only 31%, 70%, 52%, and 48%, respectively, suggesting that resting LES pressure may not fully account for reflux^[20]^. Finally, a meta-analysis of 7 studies that reported technical details of the surgical procedure and GERD symptoms or esophagitis demonstrated a transient protective effect of antral preservation, which was not preserved at longer time points^[21]^.

The effect of SG morphology on weight loss and GERD symptoms has also been investigated. In one study, upper gastrointestinal series were used to characterize SG morphology in 268 patients as Dumbbell (38%), Lower Pouch (22%), Tubular (26%), or Upper Pouch (14%). An overall increase in the prevalence of GERD symptoms, based on chart review, was observed postoperatively (68% *vs*. 48%, *P* < 0.0001), which was further characterized as *de novo* (51%), persistent (28%), worsened (58%), or resolved (14%). While sleeve morphology was associated with long-term weight loss, with the Dumbbell shape linked to less BMI reduction, no association was found between sleeve morphology and any GERD outcomes^[22]^.

Gastric tube abnormalities have been described following SG and are associated with the progression of esophagitis. In a retrospective study of 459 patients post SG who underwent pre- and postoperatively esophagogastroduodenoscopy (EGD), esophagitis was identified in 20% of the patients post SG, while gastric abnormalities were identified in 28% of the patients. The most common alterations included gastric dilation (16.1%), gastric twist (nearly 11%), neofundus (7.4%), and hiatal hernia (0.2%). Gastric tube abnormalities were associated with the progression of esophagitis (*P* = 0.013), although no significant association was found for individual abnormalities^[23]^.

Overall, the impact of LSG on factors driving reflux reflects a complex interplay of multiple mechanisms [Table 1], occurring in the context of weight loss, which can reduce the gastroesophageal pressure gradient. Additionally, the surgery itself may affect gastric motility, potentially in a protective manner, through increased gastric emptying, while other surgical effects such as disruption of the anti-reflux barrier and decrease in gastric compliance may be refluxogenic.

## PREVALENCE AND RISK OF GERD AFTER LSG

Numerous studies have examined the development of GERD or worsening of preoperative GERD after SG. However, data on the prevalence of new-onset or aggravated GERD post-LSG remain somewhat conflicting. Some studies have reported the development of *de novo* GERD^[24]^, while other studies have shown improvement in GERD symptoms^[25]^. These studies have been limited by a number of methodological differences, including the definition of GERD (defined by symptoms, endoscopic findings, and/or pH studies, often with different diagnostic cut-offs), number of patients, duration of follow-up, BMI of the patients who underwent LSG, and primary and secondary outcomes assessed.

A retrospective review of a longitudinal database of 4,832 patients who underwent LSG between 2007 and 2010 reported preexisting GERD in 44.5% of patients scheduled for SG, 84.1% with persistent GERD symptoms postoperatively, resolution of GERD symptoms in 15.9%, and 8.6% with *de novo* GERD symptoms postoperatively^[26]^. The incidence of pre- and postoperative GERD was also evaluated in a study of 162 SG patients, using questionnaires, PPI use, and upper endoscopy. GERD symptoms (68% *vs*. 34%, *P* < 0.0001) and PPI use (57% *vs*. 19%, *P* < 0.0001) increased postoperatively. Increases in Grade B (33% *vs*. 8%, *P* < 0.00901), and Grade C esophagitis (12% *vs*. 4%, *P* = 0.04) were found, with GERD symptoms in only 33%, and Grade D esophagitis were increased (9% *vs*. 0%, *P* = 0.0016) with symptoms in 57% of patients, post *vs*. pre-SG. Additionally, there was a significant increase in non-dysplastic BE (17% *vs*. 0%, *P* < 0.0001)^[16]^.

Overall, *de novo* GERD has been reported to occur in 7.4% to 58% of patients following LSG^[27]^. A systematic review and meta-analysis of 33 studies reported the prevalence of GERD symptoms (11 studies using standardized questionnaires), the use of anti-reflux medications (4 studies), and findings from esophageal function testing (3, 7, and 2 studies reported results of 24-hour pH testing, esophageal manometry, and combined pH-impedance, respectively) following LSG. A pooled risk difference for GERD before and after LSG was 4.3%. Changes in the use of anti-reflux medication could not be pooled, as some studies reported a marked increase in PPI use, while others found no change or even a decrease. Secondary outcome measures included the prevalence of new-onset GERD (reported in 24 studies) and esophagitis (4 studies). The pooled incidence of new-onset GERD was 20%, and the incidence of new-onset esophagitis ranged widely from 6.3% to 63.3%. Overall, these studies were characterized by high heterogeneity, and esophageal function testing was inconsistent^[28]^.

A prospective study utilized 24-hour transnasal pH monitoring before and after LSG to define GERD (esophageal pH < 4 for at least 4.2% of total recorded time). Preoperatively, 29 patients had normal pH studies, 86% of whom had no GERD symptoms, while 21 patients had positive pH studies, 62% of whom reported GERD symptoms. Postoperatively, 69% of patients developed a newly positive pH study, including 24% (*n* = 7) who remained asymptomatic. Among those with preexisting positive pH studies, there was no significant change in total time pH < 4 with LSG (5.9% *vs*. 7.7%, *P* = 0.296), according to the percent time cut-off criteria utilized for the study. Endoscopic findings in these patients were not reported^[29]^. Of note, GERD diagnosis is not based solely on the results of pH studies, and the significance of a positive pH study in an asymptomatic patient without evidence of mucosal injury is unclear^[11]^.

The effect of LSG and concomitant hiatal hernia repair on GERD symptoms was also evaluated in a retrospective study of 58 morbidly obese patients (average BMI 44)^[30]^. Hiatal hernia was identified in 34.5% of patients via upper gastrointestinal series, and during surgery in the remaining patients. Preoperatively, 45% of patients reported GERD symptoms or daily use of anti-reflux medication. Postoperatively, 65% of these patients remained symptomatic despite achieving > 50% reduction in excess BMI, while 35% reported resolution of symptoms. Among the 55% of patients who were asymptomatic preoperatively, most remained asymptomatic. However, nearly 16% developed *de novo* GERD symptoms postoperatively, requiring daily anti-reflux medication. Overall, LSG with concomitant hiatus hernia repair resolved preexisting GERD in only one-third of patients and was associated with new-onset GERD in nearly 16%^[30]^.

While prospective, randomized multicenter trials comparing RYGB and LSG have shown similar efficacy in terms of achieving weight loss, they have also underscored differing indications for reoperation between these procedures. Internal hernias are the primary cause of reoperations after RYGB, while severe reflux not responding to medical therapy is the leading cause of conversion from SG to RYGB^[31,32]^.

In the Swiss Multicenter Bypass or Sleeve Study (SM-Boss)^[32]^, 217 patients were randomly assigned to undergo either LSG or LRYGB, and 5-year follow-up was available for 94%. Excess BMI loss was similar between LSG and LRYGB at 5 years (61% and 68%, respectively, *P* = 0.22). Reoperation rates were comparable in frequency but differed in indication: 16% of LSG patients required reoperation, 56% of which were for severe GERD, whereas 22% of LRYGB patients required reoperation, with 39% due to internal hernias. Preoperative GERD was reported in 44% and 46% of patients randomized to LSG and LRYGB, respectively. GERD symptoms worsened more often following LSG compared to LRYGB (32% *vs*. 6%, *P* = 0.006), while symptom improvement was more frequent after LRYGB compared to the LSG group (60% *vs*. 25%, *P* = 0.002). Notably, *de novo* GERD symptoms were reported more often after LSG than after LRYGB [32% (18/57) *vs*. 11% (6/56), *P* = 0.01]^[32]^.

In the Finnish Sleeve *vs*. Bypass (SLEEVEPASS) study^[31]^, 240 patients were randomized to LSG or LRYGB, with 80% completing 5-year follow-up. While LRYGB patients had slightly greater excess weight loss compared to patients who underwent LSG, this difference did not reach statistical significance (57% *vs*. 49%). Reoperation was required in 8% of LSG patients, mostly for severe reflux (70%), and in 15% of LRYGB patients, primarily due to internal hernias (94%). Other GERD-related outcomes were not reported^[31]^.

In a non-randomized controlled trial of 75 patients undergoing SG (*n* = 35) or RYGB (*n* = 40), GERD parameters were evaluated in detail using symptoms, endoscopy, and 24-h pH monitoring both pre- and postoperatively. GERD was defined as reflux esophagitis of Los Angeles Grade ≥ B or increased total acid exposure (> 6%). Postoperatively, typical GERD symptoms, reflux esophagitis, hiatal hernia, and distal esophageal acid exposure time were all higher in post-LSG than in post-LRYGB patients. LSG patients with preoperative GERD continued to have GERD postoperatively and *de novo* GERD developed in 68% of LSG patients compared to 17% of LRYGB patients. Predictors of postoperative reflux esophagitis or GERD were LSG (OR = 12.2, 17.9), preoperative esophagitis ≥ Grade B (OR = 8.7, 7.6), and age (OR = 1.9, 2.0)^[33]^.

In addition, a recent retrospective analysis of 159 patients who underwent LSG and 183 patients who underwent LRYGB at a single institution, with 1–5 years of follow-up, showed that early complications (< 30 days) were more frequent with LRYGB than with LSG (10.4% *vs*. 3.8%). Surgical site infection was the most common early complication in both groups, but occurred more frequently in the LRYGB group than in the LSG group (6.6% *vs*. 1.9%, *P* < 0.035). On the other hand, late complications (> 30 days) were more common overall in the LSG group than in the LRYGB group (31.4% *vs*. 10.4%), with GERD being the most frequent late complication (30.8% *vs*. 7.7%, *P* < 0.001)^[34]^.

A systematic review and meta-analysis of 22 studies involving 20,495 LSG patients reported a pooled GERD prevalence of 35% (33% in observational studies and 58% in clinical trials). Although the included studies were affected by heterogeneity, the overall findings suggested a “moderate to high risk of developing GERD following LSG”^[35]^. This review shows a lower estimate than a previously conducted meta-analysis of nine studies, which found an OR of 3.61 for developing GERD following LSG, with 50% of participants affected^[36]^. The discrepancy is partly attributed to methodological differences between the studies. Regardless, both meta-analyses indicate that more than one-third of patients develop GERD following surgery, which is not a negligible number that should be clearly communicated to patients as a potential outcome. Despite variation in reported rates of *de novo* or worsening GERD and heterogeneity of the studies, the overall effect of LSG appears to be refluxogenic.

## PREVALENCE AND RISK OF BE AFTER LSG

GERD and obesity^[37]^ are both considered risk factors for BE, the only recognized precursor to esophageal adenocarcinoma. A systematic review and meta-analysis^[38]^ of 46 retrospective and cohort studies totaling 10,718 patients with 3–132 months of follow-up evaluated the primary outcomes of esophagitis and BE prevalence after LSG. Secondary outcomes included changes in *de novo* reflux. The pooled prevalence of increasing GERD after SG was 19%, including in subgroup analyses of studies reporting long-term outcomes over 24 months. The pooled prevalence of new-onset GERD was 23%, and 20% in long-term studies. Esophagitis occurred in 30% of patients in the pooled analysis and in 28% of long-term studies. The prevalence of BE was 6% overall and 8% in long-term studies. Severe reflux accounted for conversion to RYGB in 4% of patients, both in the overall pooled analysis and in the subgroup analysis of long-term studies. Studies informing these results exhibited substantial heterogeneity^[38]^.

A systematic review and meta-analysis of 10 studies^[39]^, including 680 patients who underwent upper endoscopy between 6 months and 10 years after SG, showed a pooled prevalence of *de novo* BE of 11.6%. There was no significant association between the prevalence of BE and postoperative GERD symptoms. Moreover, the risk of esophagitis increased by 13% per year following SG^[39]^. The American Society for Gastrointestinal Endoscopy recommends that populations with a 10% risk of BE should undergo screening^[40]^. While this study indicates a risk of *de novo* BE following SG, several questions remain unanswered, including whether this risk is outweighed by the metabolic benefits of weight loss, making the role of routine screening and surveillance for BE in post-SG patients unclear^[41]^.

A retrospective cohort study of a prospectively maintained database of 126 SG patients reported that 29.5% of patients had reflux esophagitis at 5-year follow-up (Grade A, 15.2%; Grade B, 11.4%; Grade C, 11.4%; and Grade D, 2.9%). BE was identified in 5.7% of patients at the 5-year mark after SG. The authors suggested long-term BE surveillance in SG patients, regardless of reflux symptoms^[42]^. Current expert opinion recommends offering BE screening three or more years after LSG^[43]^.

## PREDICTORS OF GERD POST SG

The prevalence and predictors of GERD after LSG were evaluated in 213 patients using the GERD-HRQL questionnaire administered pre- and postoperatively; objective testing was not performed. Although preoperative GERD symptoms such as heartburn and regurgitation were frequent, they could not predict the new onset or worsening of GERD symptoms postoperatively^[44]^.

A prospective cohort study of obese patients who underwent 24-hour pH monitoring (*n* = 47) and esophageal manometry (*n* = 30) preoperatively and one year after SG aimed to identify clinical and manometric factors of GERD (defined as total % of time with pH < 4 exceeding 4.2%). Among patients who developed *de novo* GERD (51%), there was a decrease in basal intragastric pressure postoperatively (17.2 ± 3.7 *vs*. 11.5 ± 3.8, *P* < 0.001), while maximal intragastric pressure following swallows increased (25.4 ± 9.4 *vs*. 49.2 ± 22.0, *P* < 0.05). LES resting pressures remained unchanged pre- and postoperatively and did not correlate with changes in pH. Overall, no clinical or manometric factors were predictive of *de novo* GERD in this study^[45]^.

More recently, there has been an attempt to objectively determine whether a preoperative GERD diagnosis was associated with worsening postoperative symptoms. A single-center case series evaluated the effects of SG on 20 patients with GERD symptoms, nine of whom had a positive pH study and eleven a negative pH study. In this small study, conversion to gastric bypass (two patients) only occurred in the cohort with a positive pH study, and no conversions were reported in those with a negative pH study. Postoperative quality-of-life scores and GERD symptoms remained similar between the two groups^[46]^.

In separate studies, preoperative esophagitis or GERD and increasing age have been identified as predictors of post-SG esophagitis or GERD^[33]^. A multicenter cohort study of 1,537 patients who underwent LSG assessed GERD using a combination of questionnaire and upper endoscopy. Nearly 25% of patients had postoperative GERD, 21% of whom had *de novo* symptoms, and 2% of whom had worsened or unchanged preoperative symptoms. Multivariate analysis identified antral preservation and gastropexy as independent protective variables against the development of postoperative GERD, while antral resection and smoking were identified as risk factors for post-LSG GERD^[47]^.

Factors associated with increased severity of erosive esophagitis one year after LSG have also been evaluated. In a retrospective review of 316 post-LSG patients^[48]^, 58% developed *de novo* erosive esophagitis, with severe cases occurring in 3%. Multiple logistic regression analyses identified male sex, hiatal hernia after LSG, and preoperative erosive esophagitis as independent risk factors for the presence of erosive esophagitis after LSG. When considering factors associated with increased severity of erosive esophagitis, male sex (OR = 2.55, *P* < 0.001) and hiatal hernia after LSG (OR = 3.17, *P* < 0.001) were identified as independent risk factors for increased severity of erosive esophagitis post LSG. Interestingly, the presence of preoperative erosive esophagitis was negatively associated with increased severity of erosive esophagitis post LSG (OR = 0.25, *P* < 0.001). Overall, the incidence of severe erosive esophagitis was low among those without significant preoperative disease^[48]^.

Demographic and manometric parameters were evaluated as possible predictors of GERD in a retrospective analysis of 164 SG patients with GERD symptoms or preoperative esophagitis who underwent high-resolution manometry^[49]^. Multivariate analysis identified preoperative GERD symptoms (OR = 2.5, *P* = 0.013), female sex (OR = 3.4, *P* = 0.002), and distal contractile integral (DCI) ≥ 1,623 mmHgscm (OR = 0.3, *P* = 0.003) as independent determinants of postoperative GERD^[49]^. While methodological limitations may apply, these results demonstrate the potential utility of thorough preoperative physiologic evaluation.

Quantification of physiologic parameters of the gastroesophageal junction impedance planimetry technology utilizing EndoFLIP^™^ has also been used to identify predictors of GERD following SG^[50]^. In a retrospective review of 28 patients who underwent robotic SG, individuals with *de novo* or worsening GERD demonstrated higher post-sleeve distensibility index (DI) and lower post-sleeve LES pressure compared to asymptomatic patients^[50]^. In contrast, a prospective pilot study of nine LSG patients, evaluated pre-, intra-, and postoperatively, demonstrated that GERD was associated with higher preoperative GEJ distensibility, while postoperative DI did not correlate with GERD^[51]^.

Overall, symptoms alone are insufficient predictors of GERD severity following LSG. It is not surprising, however, that objective evidence of preoperative GERD, particularly high-grade erosive esophagitis or a disrupted anti-reflux barrier, appears to be more prognostic of poor postoperative outcomes. Identifying patients at greatest risk for developing *de novo* GERD after LSG remains an active area of investigation and will likely benefit from studies detailing the results of comprehensive preoperative evaluation.

## PREOPERATIVE EVALUATION FOR GERD IN PATIENTS UNDERGOING SG

The need for evaluation of GERD prior to SG remains a topic of debate, with conflicting data in surgical literature regarding determining which patients should be formally evaluated and which diagnostic modalities should be used. In 2016, a publication reported findings from an international expert panel of 120 bariatric surgeons surveyed in 2014, and compared the results to an earlier expert survey from 2011 and a survey of 103 general surgeon members. Compared to the general surgeon group, fewer members of the expert panel considered GERD to be a contraindication to SG (23% *vs*. 52%, *P* < 0.001), whereas more of the expert panel considered BE a contraindication (80% *vs*. 31%, *P* < 0.001). In terms of workup for GERD, fewer experts in 2014 recommended that patients with GERD should have pH monitoring and manometry prior to surgery, when compared to 2011 (33% *vs*. 50%, *P* = 0.033). Overall, the consensus was that GERD should not be considered an absolute contraindication to SG, while BE was more strongly viewed as a contraindication by bariatric experts than by general surgeons^[52]^.

The American Society for Metabolic and Bariatric Surgery (ASMBS) updated its position statement on SG as a bariatric procedure in 2017. It suggested that, aside from expert opinion, there was limited evidence to support excluding patients with preexisting GERD from undergoing SG. Regardless of the strategy used to screen or evaluate for GERD preoperatively, counseling for GERD-related outcomes has been recommended for all patients undergoing SG^[27]^.

The ASMBS has provided an opinion on the role of upper endoscopy before and after SG. Routine preoperative upper endoscopy was justifiable as it can identify not only GERD-related complications but also actionable gastric abnormalities that may be missed in the remnant. Therefore, the decision to perform endoscopy was left to the discretion of the surgeon. Postoperative upper endoscopy was recommended in patients with gastrointestinal symptoms, including GERD, and also considered in asymptomatic patients for the detection of BE^[43]^.

A meta-analysis by Bennett *et al*. involving 48 studies and 12,261 patients showed that preoperative upper endoscopy led to changes in surgical management in 8% of cases, and medical management in 28% of cases. Overall, given that the proportion of EGD findings that resulted in significant management changes was low, the authors concluded that preoperative EGD could be considered selective and optional^[53]^. Similarly, a meta-analysis by Parikh *et al*., consisting of 28 studies and 6,616 patients, showed that preoperative upper endoscopy led to a change in clinical management in only 8% of the cases^[54]^.

Recently, comprehensive esophageal evaluation, consisting of symptom assessment, upper endoscopy, esophageal manometry, and 24-hour pH/impedance monitoring, was conducted on 500 obese patients being considered for SG, and the results were compared to data from 25 healthy volunteers^[55]^. Eighty-nine patients tested negative for GERD (no symptoms, normal upper endoscopy, normal manometry, normal pH/impedance testing) and underwent SG. Patients who tested positive for GERD underwent SG in combination with either a form of fundoplication or RYGB; however, the details were not further described. At the two-year follow-up, only two patients (2.5%) reported *de novo* GERD symptoms. Further workup revealed a middle gastric stricture and GERD symptoms resolved post endoscopic dilatation. In the 43 patients who underwent follow-up upper endoscopy, none had evidence of esophagitis or BE. These findings suggest that the risk of developing *de novo* GERD post-SG is low in those without preoperative GERD symptoms and normal comprehensive esophageal evaluations^[55]^.

Ultimately, recommendations regarding the workup of GERD have been made based on position statements that are limited by available data, and are not considered local, regional or even national standard of care. Workup tends to be driven by institutional culture rather than larger data-driven consensus. ASMBS published a position statement in 2021 to this effect^[43]^. Some experts recommend that all patients should be assessed for GERD symptoms, but do not take a firm position on whether upper endoscopy should be performed only on symptomatic patients, recognizing that upper endoscopy performed in asymptomatic patients may also impact surgical choices. Another consideration is that those with objective evidence of severe GERD, such as LA Class C or D esophagitis, AET > 6% on pH testing, or severe GERD symptoms preoperatively, may be better suited for alternative bariatric procedures.

European guidelines advocate routine upper endoscopy preoperatively. In 2020, the Federation for the Surgery of Obesity and Metabolic Disorders (IFSO)^[56]^ recommended considering upper endoscopy for all patients undergoing bariatric surgery, regardless of the presence of upper GI symptoms, due to the potential for unexpected findings that may alter management. The IFSO also recommended surveillance at 1 year post bariatric surgery and then every 2–3 years thereafter, particularly after SG, to facilitate early detection of BE^[56]^. Overall, at a minimum, experts in the US recommend a selective approach to upper endoscopy while European experts recommend routine upper endoscopy. In this regard, careful preoperative planning, including off-PPI upper endoscopy, may help identify those individuals with GERD, as symptoms have limited value for diagnosis given low sensitivity and specificity. In addition, severe esophagitis or BE, as complications of severe GERD, are considered by some to contraindicate SG^[10]^.

## ALTERNATIVE BARIATRIC INTERVENTIONS TO LSG IN PATIENTS WITH PREEXISTING GERD

The optimal surgical approach for obesity in patients with GERD remains a matter of debate. While RYGB has been widely used as the bariatric procedure of choice in obese individuals with GERD, persistent and *de novo* GERD symptoms are reported in a subset of RYGB patients^[57–59]^. A systematic review and meta-analysis comparing GERD outcomes after LSG and RYGB showed that the odds ratio of developing *de novo* GERD was higher in LSG compared to RYGB (OR = 5.10, 95%CI 3.60–7.23, *P* < 0.0001), but *de novo* GERD also occurred in 2.3% of patients post-RYGB^[60]^. In addition, longer-term follow-up studies indicate a previous underestimation of GERD symptoms post-RYGB. A Swedish population-based cohort study of 2,454 patients with RYGB and preoperative GERD symptoms (defined as use of anti-reflux medications) showed that 68% of patients required long-term treatment for reflux within 5 years^[61]^. Similarly, a prospective study of 180 RYGB patients followed for 12 years reported persistent or *de novo* GERD in 23.8%, with weight regain identified as a significant predictor of GERD on multivariate analysis (OR = 3.22, *P* = 0.029)^[62]^. A high percentage of hiatal hernia, hypotensive LES, and esophageal motility disorders have been reported in individuals with persistent GERD post RYGB^[57,58]^. The pathophysiological mechanisms underlying these symptoms seem to be multifactorial^[58,62]^ and extend beyond the scope of this review. Therapeutic management of patients with severe GERD after RYGB remains a challenge and may include modified Nissen or Toupet fundoplication, Hill procedure, or use of a magnetic sphincter augmentation device (e.g., Linx)^[58,59]^. Of course, there may be factors beyond GERD that will dictate the bariatric procedure of choice. Some patients may not even be candidates for an anastomotic type of procedure such as RYGB. In these patients, SG may be the only viable option despite the presence of GERD.

LSG combined with an anti-reflux procedure including various fundoplication techniques (posterior 360°, posterior 270°, or anterior 180°) has been proposed as an alternative approach to improve LES barrier function. A systematic review and meta-analysis of five studies (four retrospective cohort studies and one randomized, controlled trial) including a total of 539 patients (BMI ≥ 35) compared GERD-related outcomes and safety between LSG plus fundoplication (LSG + F) and LSG alone^[63]^. Overall, LSG + F resulted in improved GERD remission at the expense of less weight loss and higher postoperative complications. Major complications included gastric perforation, bleeding, leak, and dysphagia in the LSG + F group (OR = 2.56; 95%CI 1.12–5.87). The included studies were limited by the lack of objective GERD assessment, relying instead on symptoms and/or PPI use, and were marked by heterogeneity in GERD evaluation methods^[63]^. While these results suggest that LSG combined with fundoplication may be a promising alternative bariatric procedure for patients with GERD, further research is warranted to clarify the overall clinical benefit, better define the risk profile, and determine the positioning of its use in the bariatric surgery toolbox.

A number of endoscopic bariatric therapies are also available for weight loss. In patients with known GERD or its complications, some experts suggest avoiding LSG and instead favoring options such as endoscopic sleeve gastrectomy (ESG)^[64]^. Endoscopic interventions such as ESG have been shown to produce significant weight loss and improve comorbidities^[65]^. Unlike LSG, ESG is considered an “anatomy-sparing” technique, as it preserves the natural components of the EGJ uninterrupted. In this endobariatric procedure, a gastric sleeve is created by plicating the greater curvature of the stomach, from the level of the incisura to the gastric cardia^[66]^.

Results of comparative analysis of surgical (e.g., RYGB and SG) and endoscopic (e.g., ESG) bariatric interventions with respect to GERD and other outcomes are of particular interest. A meta-analysis of seven comparative studies involving 6,775 patients directly comparing ESG (*n* = 3,413) and LSG (*n* = 3,362)^[66]^ showed that ESG achieved clinically meaningful total body weight loss (TBWL) percentages at 6, 12, and 24 months, although these were lower than those achieved with LSG (15.2% ± 6.3%, 19.1% ± 7.9%, and 16.4% ± 10.1% *vs*. 18.8% ± 7.5%, 28.9% ± 8.2%, and 22.3% ± 8.3%, respectively; *P* < 0.0001). The incidence of adverse events was lower with ESG compared to LSG (0.7% *vs*. 1.7%), although this difference did not reach statistical significance. Importantly, the incidence of new-onset GERD was significantly lower following ESG compared to LSG (1.3% *vs*. 17.9%, RR = 0.10, *P* = 0.006)^[66]^. Overall, these studies support the consideration of ESG as an alternative bariatric option, particularly in patients with mild-to-moderate obesity. However, it is important to note that only observational studies were included in this meta-analysis. Thus, prospective, and preferably RCTs, are needed to validate these results. Overall, in patients with preexisting GERD for whom LSG should not be offered, several alternative bariatric surgical options can be considered [Table 2].

## EVALUATION AND MANAGEMENT OF GERD POST-LSG

The evaluation and management of GERD after bariatric surgery remains an active area of investigation and is guided by expert opinion. A group of 46 recognized bariatric and metabolic surgical experts from 25 countries participated in a Delphi consensus to provide an algorithm for clinical decision-making in cases under consideration for revisional surgery after SG for insufficient weight loss, weight regain, and GERD^[67]^. The consensus was that multidisciplinary evaluation (91.3% agreement) along with at least 12 months of medical and supportive management (73.9% agreement after round 2) should be performed prior to reoperation for GERD after LSG. Most experts agreed on the importance of upper endoscopy (95.6% agreement) and upper GI series (82.6% agreement) prior to reoperation, although consensus was not reached regarding additional diagnostic evaluation such as pH monitoring or esophageal manometry, despite two rounds of voting. In cases of symptomatic GERD after SG and adequate weight loss, the consensus was that continuing medical treatment for at least 1–2 years (86.9% agreement) and RYGB (97.7% agreement) were acceptable. Despite two rounds of voting, consensus could not be reached regarding the suitability of magnetic sphincter augmentation with LINX. In cases of symptomatic GERD after SG and inadequate weight loss or with weight regain, recognizing the contribution of persistent obesity to GERD symptoms, 97.7% agreed that RYGB was an acceptable option. Finally, in cases of symptomatic GERD after SG and excessive weight loss, 97.8% agreed that RYGB was an acceptable option. Cruroplasty/HH repair was considered acceptable by nearly 57% of experts, but consensus could not be reached despite two rounds of voting. Similarly, magnetic sphincter augmentation was considered an acceptable option by up to 53.3% of experts in cases of symptomatic GERD after SG and excessive weight loss, but no consensus was reached.

Overall, when considering revisional surgery following LSG, reevaluation with EGD or upper GI series was widely regarded as warranted, while controversy remained regarding whether additional testing was essential. In addition, in cases where medical therapy was deemed inadequate, the only revisional surgical approach to achieve expert consensus was RYGB^[67]^. Studies informing the importance of medical management and evidence supporting the escalation to additional endoscopic or surgical intervention post-LSG such as magnetic sphincter augmentation or revisional RYGB are described below.

In individuals who develop *de novo* reflux after LSG with adequate weight loss, expert consensus supports that the use of medical management^[67]^ is appropriate and reasonable, typically with PPIs as described in guidelines for GERD management^[68]^. In fact, medical therapy has proven to be quite effective at managing GERD symptoms post LSG. A multicenter cohort study of 379 LSG patients who had GERD postoperatively^[47]^ found that, after excluding postoperative anatomic abnormalities as the etiology of symptoms, medical treatment with PPIs was effective in 79% of cases (*n* = 300). All patients experienced resolution of symptoms within two years, allowing for discontinuation of the PPI^[47]^.

More recently, potassium-competitive acid blockers (PCABs) such as vonoprazan have become available for the treatment of both erosive and nonerosive GERD. A randomized controlled trial of 1,024 adults with erosive esophagitis demonstrated that vonoprazan 20 mg daily was noninferior, and superior to the PPI lansoprazole 30 mg daily for healing of erosive esophagitis, particularly in the patient group with severe esophagitis (LA Grades C/D). The study also demonstrated that vonoprazan 20 or 10 mg was noninferior, and superior to lansoprazole 15 mg for maintenance of healing^[69]^. The safety profile of vonoprazan over 5 years has recently become available. In a cohort of Japanese patients who achieved healing of erosive esophagitis with either vonoprazan 20 mg daily (*n* = 139) or lansoprazole 15 mg daily (*n* = 69), participants were then placed on maintenance therapy with vonoprazan 10 mg daily or lansoprazole 15 mg daily for a duration of 260 weeks. Although serum gastrin levels were higher in those treated with vonoprazan, there were no reports of malignant alterations or neuroendocrine tumors in either group. The incidence of adverse events was also similar between these groups^[70]^. While longer-term studies assessing its efficacy and cost-effectiveness have not yet been performed, and issues with affordability and insurance coverage for vonoprazan make its widespread use unclear, PCABs represent a promising option particularly in cases of severe esophagitis post-LSG.

Regurgitative GERD following LSG, in particular, may not be responsive to PPIs. In patients who have achieved adequate weight loss post-LSG but with severe GERD, augmentation of the LES with the laparoscopically placed magnetic sphincter device Linx may be an option as a rescue therapy. However, as mentioned above, consensus regarding this approach has not been reached by expert surgeons^[67]^. A retrospective analysis evaluated 22 post-LSG patients with persistent GERD symptoms requiring daily anti-reflux medications who subsequently underwent laparoscopic MSA. The mean interval time to placement was 43.6 months, with follow-up ranging from 6 to 12 months^[71]^. No adverse events including erosions or device-related mortality were reported. GERD-HRQL scores improved significantly following MSA (from 43.8 to 16.7, *P* < 0.0001), and most patients (82%) were able to discontinue anti-reflux medications (*P* < 0.049)^[71]^.

An observational, multicenter single-arm prospective study evaluated the safety and efficacy outcomes of MSA in 30 LSG subjects with persistent GERD symptoms for > 6 months requiring daily PPI^[72]^. Patients were followed for 12 months post-implantation. Dysphagia and pain occurred in 6.7% of participants but self-resolved. Two subjects required device removal, one at 2 weeks and another at 4 months. GERD-HRQL scores improved by 81% (*P* < 0.001), PPI usage decreased by 96% (*P* < 0.001), and total distal acid exposure time normalized or decreased by more than 50% in 44% of patients (from 16.2% at baseline to 11% at 12 months, *P* = 0.028)^[72]^. While these results are promising, larger, well-designed prospective or randomized controlled trials are still needed to evaluate long-term safety, efficacy, and cost-effectiveness. Although there is no consensus on its use post-LSG, magnetic sphincter augmentation with LINX may be considered a rescue option in carefully selected candidates.

Radiofrequency therapy administered via the Stretta system was approved by the FDA in 2000 for the treatment of GERD. However, systematic reviews and meta-analyses for the efficacy of radiofrequency therapy on the LES have yielded inconsistent results with regard to its effectiveness in treating GERD^[73,74]^. The available data on bariatric patients are equally unsatisfying. A retrospective review of 15 patients with GERD symptoms following LSG evaluated outcomes after Stretta therapy^[75]^. In this small cohort study, mean HR-QoL scores were unchanged between pre- and post-Stretta at 6 months. PPI use was discontinued in 20% of patients and reduced from twice to once a day in 27%. However, most patients (67%) remained dissatisfied with the results. Importantly, there was one case of hematemesis post-Stretta, requiring re-endoscopy^[75]^. Overall, the efficacy of Stretta remains uncertain even in the bariatric population.

Other alternatives that have been explored in smaller studies and case presentations include electrical stimulation of the LES^[76]^ and endoscopic anti-reflux mucosectomy (ARMS), wherein scar tissue is generated at the level of the EGJ via EMR/ESD^[77]^. The safety and efficacy of LES electrical stimulation were investigated in a multicenter study involving 17 post-SG patients with GERD symptoms partially responsive to PPI across six centers^[76]^. The median follow-up was 12 months. Significant improvement was observed in median GERD-HRQL scores post-procedure (*P* < 0.001), and distal esophageal acid exposure decreased from 13.2% to 5.8% (*P* = 0.01). At the final follow-up, 41% had completely discontinued PPIs^[76]^. A case report of ARMS^[78]^ described a 71-year-old female who underwent SG in 2013 and presented worsening GERD symptoms despite twice-daily PPI use. Her primary symptom was regurgitation. She was not a candidate for RYGB due to comorbidities including COPD and dilated cardiomyopathy. At 7-month follow-up post-ARMS, she had improvement in her GERDQ score from 11 to 8, and normalization of pH testing using DeMeester scores (17.7 to 5.8). Acid exposure time was not reported in this study^[78]^. While evidence remains limited, these procedures may hold promise for the management of bariatric patients. In particular, for patients who are either unwilling or medically unsuitable to undergo RYGB, continued exploration into these alternative approaches is warranted.

As previously noted, in cases where medical therapy is inadequate, the only revisional surgical approach to achieve consensus among experienced bariatric surgeons in the Delphi process was RYGB. An earlier meta-analysis and systematic review examining GERD outcomes following LSG and RYGB included four studies that reported conversion rates, with the proportion of conversion from LSG to LRYGB for GERD ranging from 1.82 to 8.91%^[60]^. Improvement and/or resolution of GERD symptoms has also been investigated with revisional RYGB following LSG. A retrospective, 3:1 propensity score-matched cohort study compared outcomes between primary RYGB (*n* = 332) and conversional RYGB following LSG (*n* = 149), with a 2-year follow-up^[79]^. GERD (LA Classification Grade A-D esophagitis) with or without weight loss failure, was the indication in 30% of conversional RYGB cases^[79]^. Although symptoms were not reported, post-conversional RYGB revealed LA Grade A esophagitis in 7.4% of patients^[79]^, which does meet current diagnostic criteria for GERD^[80]^. Notably, no cases of *de novo* GERD were reported in this study during the follow-up of only 2 years. While it is clear that in a subset of patients post-primary RYGB, GERD symptoms either occur *de novo* or do not resolve^[59]^, this cohort study^[79]^ supports the Delphi consensus agreement^[67]^ to offer LSG patients with medically refractory GERD a conversion to a RYGB [Table 3].

## CONCLUSIONS

**Figure 1.**
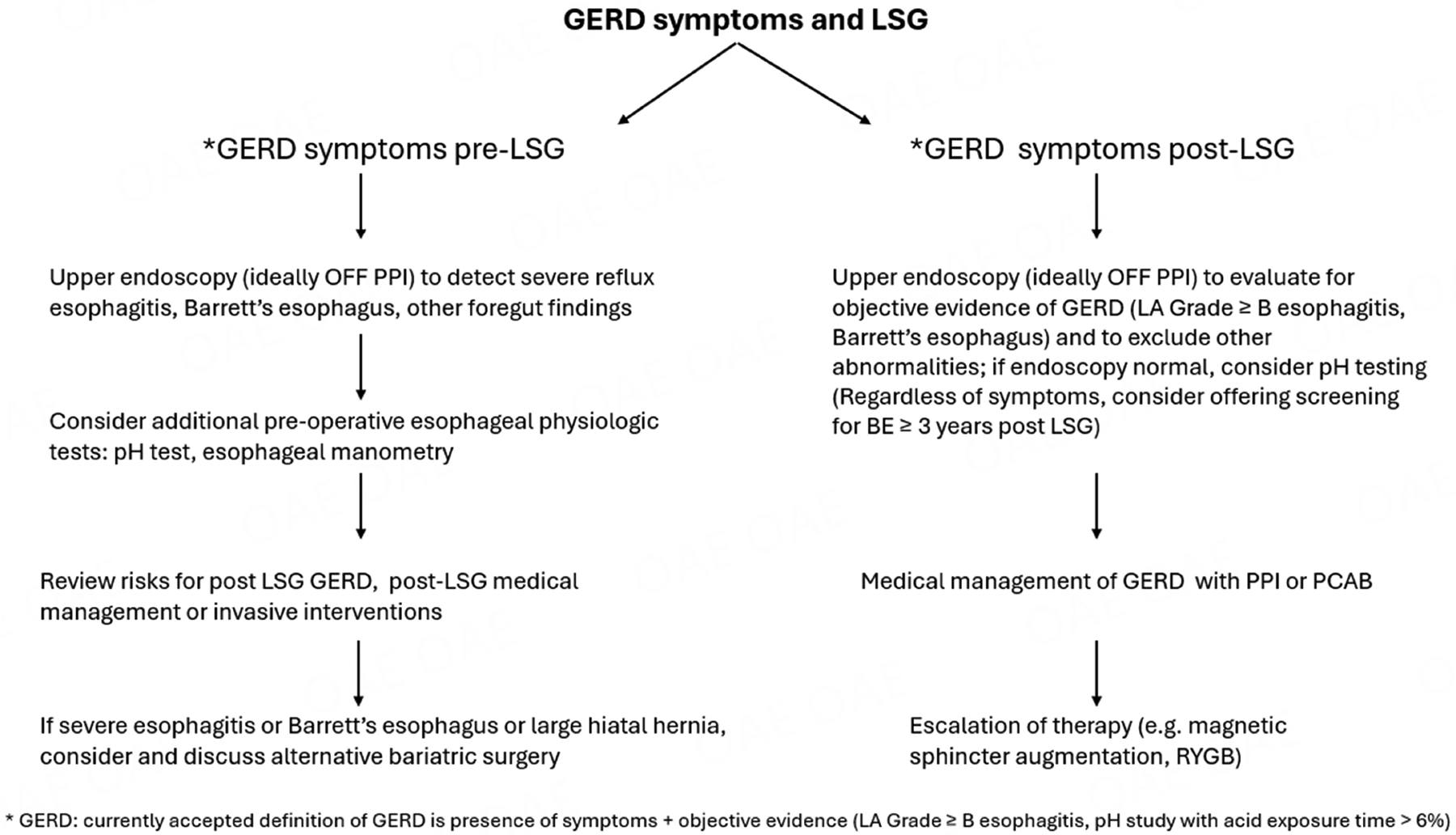
Proposed approach to GERD in patients undergoing LSG. GERD: Gastroesophageal reflux disease; LSG: laparoscopic sleeve gastrectomy.

GERD and obesity coexist in many patients, and the prevalence of both conditions continues to rise. One of the most common methods for treating obesity is LSG, which is effective for inducing weight loss, improving associated comorbidities, and enhancing quality of life. However, LSG is also associated with an increased risk of *de novo* and/or worsening GERD. The mechanisms leading to worsening and/or *de novo* GERD post LSG are multifactorial and may, in certain instances, be obviated by a surgical approach. Careful selection of patients for LSG through preoperative evaluation for GERD may avoid the risk of severe GERD following the procedure, although the best approach (upper endoscopy alone or coupled with additional testing) has yet to be defined. Patients with preexisting GERD, particularly those with severe esophagitis or large hiatal hernias, should be counseled about the increased risk of GERD worsening following LSG. In such cases, they should be considered a priori for alternative weight loss/bariatric options such as RYGB, or LSG in conjunction with hiatal hernia repair or fundoplication, with a discussion of the associated benefits and risks. For those patients who develop *de novo* or worsening GERD post-LSG, management should be based in part on the success of weight loss and the severity of GERD. Consensus-supported treatment options include medical management, which now encompasses both PPIs and PCABs. For suitable and willing candidates, conversion to RYGB remains a surgical option. Potentially alternative and currently exploratory approaches such as magnetic sphincter augmentation or LES sphincter stimulation could be considered as rescue options. Ultimately, precise phenotyping and careful patient selection are critical to minimizing, and/or at the very minimum, appropriately addressing the risk of *de novo* or worsening GERD following LSG [Figure 1].

## Figures and Tables

**Table 1.** Proposed mechanisms driving GERD post LSG

Imbalance between increased intragastric and decreased LES pressure (change in angle of His, decrease in basal LES pressure, and increase in intragastric pressure)
Increase in rates of hiatus hernia
Decreased gastric compliance
Surgical technique (e.g., antral preservation)

GERD: Gastroesophageal reflux disease; LSG: laparoscopic sleeve gastrectomy; LES: lower esophageal sphincter.

**Table 2.** Alternatives to LSG in obese patients with GERD

RYGB
LSG combined with an anti-reflux procedure (e.g., posterior or anterior fundoplication)
ESG

LSG: Laparoscopic sleeve gastrectomy; GERD: gastroesophageal reflux disease; RYGB: Roux-en-Y gastric bypass; ESG: endoscopic sleeve gastrectomy.

**Table 3.** Interventions in post-LSG patients with GERD

Aggressive medical management with PPI or PCAB
Laparoscopic magnetic sphincter augmentation with LINX
Laparoscopic RYGB
Other alternatives that have not shown efficacy or are not well studied: radiofrequency therapy to the LES (Stretta), ARMS

LSG: Laparoscopic sleeve gastrectomy; GERD: gastroesophageal reflux disease; PPI: proton pump inhibitor; PCAB: potassium-competitive acid blocker; RYGB: Roux-en-Y gastric bypass; LES: lower esophageal sphincter; ARMS: anti-reflux mucosectomy.
